# Chitosan and chitooligosaccharide utilization in phytoremediation and biofortification programs: current knowledge and future perspectives

**DOI:** 10.3389/fpls.2014.00616

**Published:** 2014-11-12

**Authors:** Marta W. Vasconcelos

**Affiliations:** CBQF - Centro de Biotecnologia e Química Fina - Laboratório Associado, Escola Superior de Biotecnologia, Universidade Católica Portuguesa/PortoPorto, Portugal

**Keywords:** biofortification, chitooligosaccharide, chitosan, mineral, phytoremediation

## Introduction

Chitosans and chitooligosaccharides (CHOS) are polysaccharides with a broad range of applications, including plant growth promoting activities. Both compounds have also been shown to have metal chelating properties. However, several factors, such as degree of polymerization, degree of deacetylation, pH, temperature, concentration, application method, viscosity and purity, can influence chitosan and CHOS mode of action. Most applications of chitosans and CHOS so far have been in the biomedical industry. But given that there is a great interested to find novel ways to enhance the plant's nutritional value (biofortification) or to modulate the capacity of plants to extract toxic metals from the soil (phytoremediation), a better understanding of chitosan and CHOS effects in plant systems is warranted.

### What are chitosans and CHOS?

Before discussing the possible applications of these molecules, it is important to clarify what they are. Chitin, chitosans and CHOS are three inter-related compounds of polysaccharidic nature. Chitin was first isolated in 1811 (Domard and Domard, 2002), and it is the second most important biopolymer in the world after cellulose (Rinaudo, 2006). Even though its first isolation was in mushrooms, chitin has since been obtained from many other sources, including cell walls of several yeast and fungal strains and from the exoskeleton of crawfish, shrimp and crabs. It is composed of a long-chain homopolymer of *N-acetyl-D-glucosamine* (GlcNAc), (1–4)-linked 2-acetamido-2-deoxy-β-D-glucan (Park and Kim, 2010) and is insoluble in water, limiting its utilization in living systems (Park and Kim, 2010). It can, however be broken down into other molecules with higher solubility. Chitosan is one of such molecules. It is at least 50% deacetilated and it is aqueous in acidic media (Rinaudo, 2006; Aam et al., 2010). CHOS have a degree of polymerization higher than 20% and an average molecular weight less than 3900Da (Lodhi et al., 2014). Still, chitosans and CHOS can be very diverse in several features such as (i) sequence, (ii) degree and pattern of *N*-acetylation; (iii) fraction of *N-*acetylated residues; (iv) degree of polymerization; (v) molecular weight, and polydispersity. This vast degree of compositional variability is in fact one of the major obstacles in chitosan research. Comprehensive knowledge on their mode-of-action is still scarce, because most published studies are done with heterogeneous mixtures and because production of well-defined chitosans and CHOS is not easily done (Montilla et al., 2013). Further, chitosan is easily degraded into CHOS by naturally occurring hydrolytic enzymes in most living systems, including plants. Thus, some of the attributed functions to chitosans may in fact be due to CHOS. Despite these issues, there has been a recent interest in using these compounds to stimulate plant growth (Abu-Muriefah, 2013) and to modulate plant mineral concentrations (Chatelain et al., 2014).

### Can they be used for phytoremediation or biofortification?

**Figure 1:**
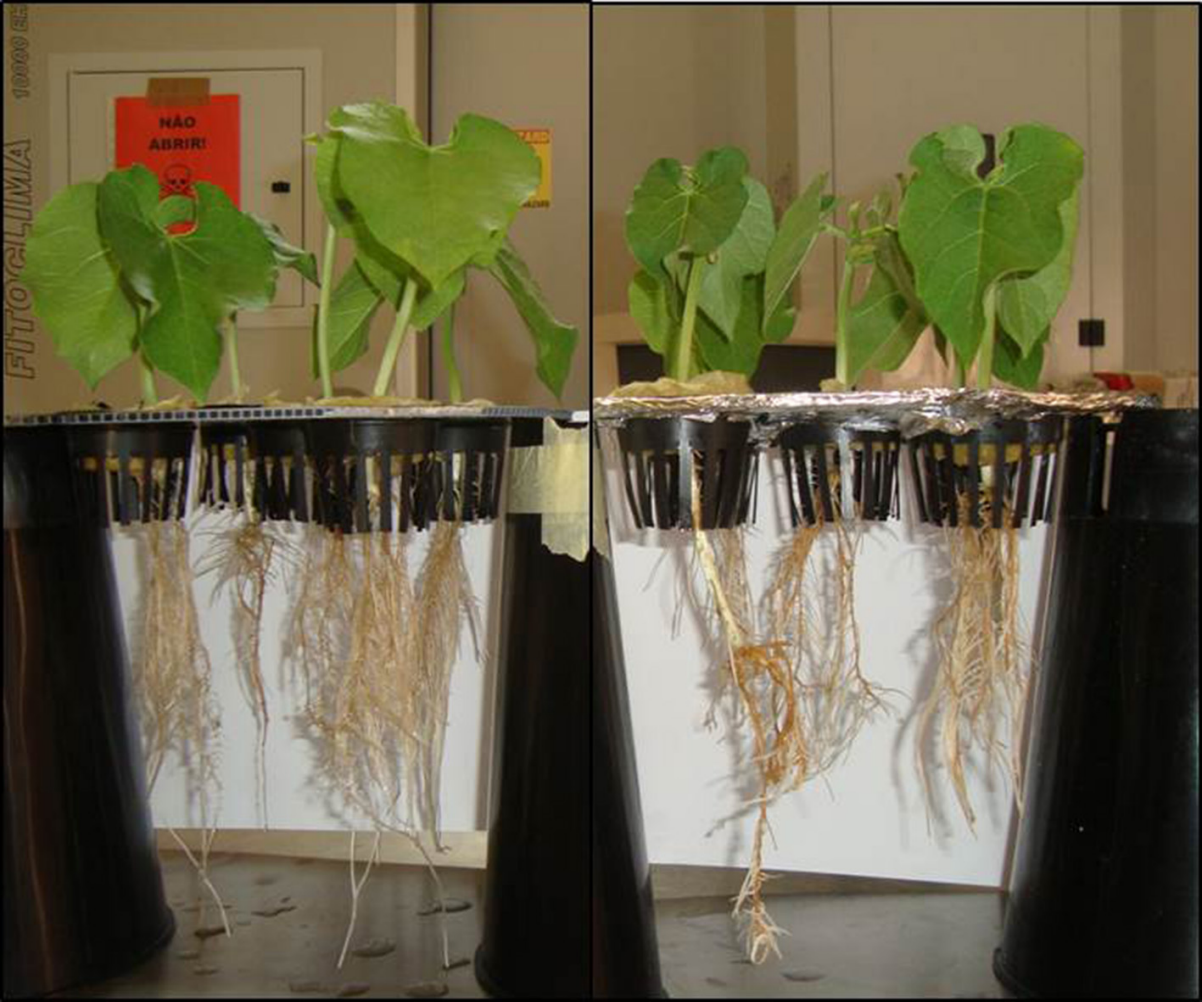
**Common bean (*P. vulgaris* L.) plants grown 12 days hydroponically without (left) or with (right) 0.01 g/L CHOS**.

The most commonly reported utilizations of chitosans and CHOS have been in the human biomedical industry (Park and Kim, 2010), as both compounds seem to possess medicinal properties (Mourya et al., 2011). On the other hand, the applications of CHOS and chitosan in plant science have been less diverse, and mostly linked to their antimicrobial activity. Both molecules are known to induce the biosynthesis of several antimicrobial compounds, namely phytoalexins (Vasyukova et al., 2001), callose (Ren et al., 2001) and lignin (Barber et al., 1989). Several papers have reported that chitosans and CHOs can be used, for example, to enhance the plant's defense against bacteria (Tikhonov et al., 2006; Rabea and Steurbaut, 2010), fungi (Park et al., 2002; Trotel-Aziz et al., 2006) and nematodes (Khalil and Badawy, 2012; Nunes da Silva et al., 2014). Besides plant defense, a broader range of influences in plant growth and development have been shown. They have also been used to augment foliar biomass in lettuce (Benavides-Mendoza et al., 2001), promote shoot and root growth in Daikon radish (Tsugita et al., 1993), hasten flowering time and increase flower number in passion fruit (Utsunomiya and Kinai, 1994), promote *in vitro* growth of cabbage (Hirano, 1988), enhance growth and hastened flowering time of *Eustoma grandiflorum* (Ohta et al., 1999), and alleviate iron deficiency chlorosis (IDC) symptoms in soybean (Vasconcelos, *unpublished data*). Abu-Muriefah (2013) suggested that foliar application of chitosan could be a promising strategy to reduce water stress symptoms in common bean, having positive effects in terms of growth and yield. When applied hydroponically, CHOS can also alter root length, mineral accumulation and shoot biomass in common bean (Chatelain et al., 2014) (Figure 1).

It is possible that these positive effects may be linked to the ability of chitosans and CHOS to chelate minerals and other nutrients, making them more available for uptake by the plant. This is important, since crop production is many times limited by low phytoavailability of essential mineral elements (White and Brown, 2010). In fact, the capacity of chitosans and CHOS to chelate certain ions makes them interesting compounds to be used in phytoremediation strategies. For example, mechanically disassembled chitosan nanofibrils have been used as metal ion chelating agents (Liu et al., 2014), and were effective chelating agents for copper (Cu), lead (Pb) and cadmium (Cd). Similarly, chitin in the form of micro-particles in solution showed a good sorption capacity for Cd, nickel (Ni), Cu, zinc (Zn), Pb and chromium (Cr) (Liu et al., 2013), suggesting their use for heavy metal treatment. Chen et al. (2011) reported on the successful biosorption of Cu, Zn, Ni, and Pb ions by cross-linked metal-imprinted chitosans and suggested their use for metal removal. Recently, an innovative bioremediation strategy using a bacterial consortium entrapped in chitosan beads was developed for oil-contaminated mangrove sediments (Angelim et al., 2013), showing promising results.

In 2011, Kamari et al. (2011a) showed that chitosans could bind silver (Ag), Zn, Cd, and Pb. In this same study they concluded that the main binding mechanism of chitosans to the metals was via their functional groups (amino and hydroxyl), and that even at very high dilution rates, the chitosans were able to retain metal ions on their surfaces. They also showed, in a separate study, that remediation of metal contaminated soil using chitosan and cross-linked treated chitosans as soil amendments is feasible (Kamari et al., 2011b). The following year, in a greenhouse pot experiment, Kamari et al. (2012) reported on the impact of chitosan application on metal accumulation in *Lolium perenne* (perennial ryegrass) and *Brassica napus* (rapeseed).

Heavy metals and beneficial minerals share many regulatory mechanisms for absorption, translocation and accumulation (Clemens et al., 2013). Thus, if chitosans and CHOS can help plants take up higher concentrations of toxic elements, it is possible that they can also increase absorption of essential minerals. Plants require 14 elements for their nutrition: nitrogen (N), phosphorus (P), potassium (K), calcium (Ca), magnesium (Mg) sulfur (S), chlorine (Cl), boron (B), iron (Fe), manganese (Mn), Cu, Zn, Ni, and molybdenum (Mo). Some of these are known to be adsorbed by chitosans and CHOS, forming interesting chelator complexes with these elements. Fe and Zn are two of the most important mineral elements whose dietary deficiency affects more than 30% of the world's population (Vasconcelos et al., 2003), thus an impact on the accumulation of these two elements would be particularly important.

Given the promising results of chitosans and CHOS at chelating toxic minerals, and since heavy metals oftentimes share, as stated before, common assimilation pathways with beneficial minerals, it is possible that chitosans and CHOS could have an application in biofortification programs. However, despite the relatively large amount of studies on bioremediation, very few studies so far have validated the use of either chitosan or CHOS in modulating metal uptake, transport and storage in plants, namely for phytoremediation purposes. Recently, we conducted a hydroponic experiment supplying different types of CHOS to *Phaseolus vulgaris*, and we looked at mineral accumulation potential for both beneficial and toxic metals (Mo, B, Zn, P, Pb, Cd, Mn, Fe, Mg, Ca, Cu, Na, Al, and K) in leaves, stems and roots (Chatelain et al., 2014). Our results showed a high variability in metal concentration with regards to tissue type and CHOS concentration. Also, despite observing positive effects, for example for K and Ca, we also observed no alterations in concentration for other elements, or even a reduction in accumulation. One downfall was that we observed toxicity of CHOS when applied at higher doses, which could be related to changes in anatomical features of the roots (Figure 1).

If targeting biofortification, the right dosage of chitosan or CHOS must be carefully chosen, and experiments must be addressed over the entire plant life cycle. However, there is no available study looking at the effect of chitosans or CHOS on seed minerals. Given the evidence thus far, this would be an interesting avenue to investigate, but scientists must be very careful when designing the experiment, as considerations of toxicity must be taken into account. Also, it is important to utilize very well characterized types of chitosans or CHOS, because their chemical composition will impact their structure and solubility. This, in turn, will directly influence their availability, stability and permanence in solution or soil matrixes.

Currently, no comprehensive studies are available on the changes on chitosan structure in the soil, but it is very likely that it will undergo major conformational and structural changes depending on soil pH. It is well known that chitosan is only soluble in acidic conditions, thus in alkaline soils, which account for 30% of total arable land, it would probably be poorly available for plant utilization. Also, it is necessary to understand if the chitosan:mineral and CHOS:mineral complex can actually enter the plant and deliver the minerals to edible plant parts, such as shoots and roots. The mobility of the complex in the plants vascular system has not been demonstrated yet, and it would provide some valuable information on the potential of this special class of molecules on depleting toxic metals from the soil, or enhancing favorable mineral accumulation by the plant.

In any case, there is enough compelling evidence that chitosans and CHOS have an impact on plant growth and development, probably due to their chelating capacities, making them more or less available to the plant. Perhaps better vehicles for delivery can be designed, similarly to the ones used for biomedical applications, before the real impact of chitosans or CHOS on plant nutrition and health can be maximized.

### Conflict of interest statement

The author declares that the research was conducted in the absence of any commercial or financial relationships that could be construed as a potential conflict of interest.
